# Lessons Learned From the Resilience of Chinese Hospitals to the COVID-19 Pandemic: Scoping Review

**DOI:** 10.2196/31272

**Published:** 2022-04-06

**Authors:** Jack Stennett, Renyou Hou, Lola Traverson, Valéry Ridde, Kate Zinszer, Fanny Chabrol

**Affiliations:** 1 Centre Population et Développement Institut de Recherche pour le Développement Université de Paris Paris France; 2 Centre de Recherche en Santé Publique Université de Montréal Montréal, QC Canada

**Keywords:** COVID-19, pandemic, SARS-CoV-2, health care, hospitals, health care strategy, hospital resilience, interventions, crisis response, crisis preparedness, public health

## Abstract

**Background:**

The SARS-CoV-2 pandemic has brought substantial strain on hospitals worldwide; however, although the success of China’s COVID-19 strategy has been attributed to the achievements of the government, public health officials, and the attitudes of the public, the resilience shown by China’s hospitals appears to have been a critical factor in their successful response to the pandemic.

**Objective:**

This paper aims to determine the key findings, recommendations, and lessons learned in terms of hospital resilience during the pandemic; analyze the quality and limitations of research in this field at present; and contribute to the evaluation of the Chinese response to the COVID-19 outbreak, building on a growing literature on the role of hospital resilience in crisis situations.

**Methods:**

We conducted a scoping review of evidence on the resilience of hospitals in China during the COVID-19 crisis in the first half of 2020. Two online databases (the China National Knowledge Infrastructure and World Health Organization databases) were used to identify papers meeting the eligibility criteria. After extracting the data, we present an information synthesis using a resilience framework. Articles were included in the review if they were peer-reviewed studies published between December 2019 and July 2020 in English or Chinese and included empirical results pertaining to the resilience of Chinese hospitals in the COVID-19 pandemic.

**Results:**

From the publications meeting the criteria (n=59), we found that substantial research was rapidly produced in the first half of 2020 and described numerous strategies used to improve hospital resilience, particularly in three key areas: human resources; management and communication; and security, hygiene, and planning. Our search revealed a focus on interventions related to training, health care worker well-being, eHealth/telemedicine, and workplace organization, while other areas such as hospital financing, information systems, and health care infrastructure were less well represented in the literature. We also noted that the literature was dominated by descriptive case studies, often lacking consideration of methodological limitations, and that there was a lack of both highly focused research on specific interventions and holistic research that attempted to unite the topics within a resilience framework.

**Conclusions:**

We identified a number of lessons learned regarding how China’s hospitals have demonstrated resilience when confronted with the SARS-CoV-2 pandemic. Strategies involving interprovincial reinforcements, online platforms and technological interventions, and meticulous personal protective equipment use and disinfection, combined with the creation of new interdisciplinary teams and management strategies, reflect a proactive hospital response to the pandemic, with high levels of redundancy. Research on Chinese hospitals would benefit from a greater range of analyses to draw more nuanced and contextualized lessons from the responses to the crisis.

## Introduction

Since the emergence of the initial outbreak in Wuhan, the SARS-CoV-2 pandemic has created serious problems for hospital resilience globally [1], with overoccupation of intensive care unit beds [2], overworking of medical staff while treating patients with COVID-19 [3], and an inability to provide other essential services [4]. In addition to the challenges of meeting increased capacity needs, health care systems and hospitals have had to prepare for and minimize the risk of nosocomial infection, which has often required major infrastructure and organizational changes [5].

The response of hospitals in China to the pandemic in early 2020, particularly the situation in Wuhan, has been well publicized. As Wuhan was the source of the first major documented nosocomial outbreak, many feared that hospitals in the city and elsewhere in China would struggle to cope with the shock of the pandemic [6]. However, hospital strategies were part of a concerted national effort, including a strict lockdown in Wuhan and forceful restrictions on movement and association, that allowed case numbers to become negligible by late March. The final patient with COVID-19 associated with the initial outbreak in Wuhan was finally discharged on June 5, 2020 [7]. Although China maintained certain restrictions throughout 2020, experienced other minor outbreaks, and suffered economic losses in the first half of the year, the country’s response has generally been viewed as a success story [8].

Defined as a system that can adapt its functioning to absorb a shock and, if necessary, transform to recover from adverse events, resilience has become an increasingly common concept within international health and development literature [9]. However, the concept is used less frequently when considering hospital and health system issues in the Chinese context. For example, in a scoping review examining resilience in disaster health management, health infrastructure safety, disaster preparedness, and medical response capability in China, Zhong et al [10] found that the topic was poorly covered in the Chinese context in both English- and Chinese-language literature.

Research by the same authors [11] led to the development of a quantitative conceptual framework of hospital disaster resilience that highlights the role of hospital resilience in the first severe acute respiratory syndrome pandemic in 2003, and a related study [12], based on questionnaires addressed to tertiary hospitals across Shandong province, identified four key factors that reflected the overall levels of disaster resilience (hospital safety, disaster management mechanisms, disaster resources, and disaster medical care capability) and compared the extent to which hospitals in the region met these criteria. They found that there was substantial variability within the province under study (Shandong) based on the type and location of hospitals. Although some elements of resilience were commonly achieved (38/41, 93% of hospitals had infectious disease surveillance), others were only managed by certain hospitals (eg, only 5/41, 12% of hospitals were able to surge staff capacity).

This literature must be reconsidered in the light of the recent SARS-CoV-2 outbreak, where the resilience of China’s hospitals has been challenged by a more severe health crisis. Although the success of China’s strategy has been attributed to achievements of the government, public health officials, and the attitudes of the public [8], the specific role of hospital resilience in this strategy is less documented. We have therefore conducted a scoping review to identify and synthesize the literature regarding the resilience of China’s hospitals in the context of the COVID-19 pandemic during the first wave and to draw lessons from these experiences to better inform and improve responses to the current pandemic and to future crises.

## Methods

### Rationale

As part of a multidisciplinary team, and with the support of two external librarians, we chose a scoping review to enable us to synthesize, with rigor and in a relatively short period of time, the state of knowledge regarding our research question, to clarify the concept of hospital resilience in the literature, and to identify and analyze relevant knowledge gaps [13]. A scoping review was preferred to a full systematic review as our goal was to provide, from a broad search, rapid information for public decision makers, stakeholders, and researchers regarding insights into hospital resilience in China. We conducted our review based on the PRISMA (Preferred Reporting Items for Systematic Reviews and Meta-Analyses) methodology specific to scoping reviews, which is largely based on the methodological framework of Arksey and O’Malley [14].

### Protocol and Registration

In June 2020, we designed a protocol in advance of the study and published it on protocols.io [15].

### Relevant Literature Identification

We conducted a systematic search using two different strategies to select appropriate academic literature from each context.

For the English-language literature, we have based our research on a collection of articles related to the COVID-19 pandemic published on the World Health Organization (WHO) website [16]. These articles were collected from the following databases: Medline (Ovid and PubMed), PubMed Central, Embase, CAB Abstracts, Global Health, PsycInfo, Cochrane Library, Scopus, Academic Search Complete, Africa Wide Information, CINAHL, ProQuest Central, SciFinder, the Virtual Health Library, LitCovid, WHO COVID-19 website, Centers for Disease Control and Prevention (CDC) COVID-19 website, China CDC Weekly, Eurosurveillance, Homeland Security Digital Library, ClinicalTrials.gov, bioRxiv (preprints), medRxiv (preprints), chemRxiv (preprints), and SSRN (preprints).

The search terms included the following keywords, comprising the three concepts: (1) China; (2) health care systems, hospitals, and professionals; and (3) resilience. English-language search terms (see Multimedia Appendix 1) and the search methods were checked by a professional librarian affiliated with the Centre Population et Développement. We searched for relevant Chinese-language articles on the database China National Knowledge Infrastructure (CNKI) using the search terms found in Multimedia Appendix 2. The request in Chinese was designed in consultation with a Chinese-speaking librarian from the Bibliothèque universitaire des langues et civilisations in Paris.

To limit the results to peer-reviewed journals, we limited the search on CNKI to five subcategories: those included in the *Science Citation Index*, the *Engineering Index*, the Beidahexin (Beijing University Core Journal Database), the Chinese Social Science Citation Index, and the Chinese Social Science Database.

The selection of evidence sources was conducted following an extended iterative process, confirming that there was complete overlap of articles with searches on other platforms (eg, Wanfang, Google Scholar, PubMed, or CDC website).

### Data Extraction Process

The following information was extracted from each of the selected articles: title, authors, publication type, type of resilience, whether resilience was explicitly referred to, the hospital dimension, main objectives of the article, a slightly adapted Mixed-Methods Appraisal Tool (MMAT) evaluation, a simple representation of the results, limitations and main findings, recommendations by the authors, and some subjective notes by the reviewers. The MMAT was adapted to better capture single case studies [15].

### Study Selection

Articles were included in the review if they were published between December 2019 and July 2020, were published in English or Chinese, focused on the resilience of Chinese hospitals in the COVID-19 pandemic, included empirical results, included accessible full articles, and were not considered gray literature (eg, press articles, letters, or editorials). Two reviewers (anonymous) used the software Rayyan [17] to select the papers using a two-stage review process.

### Reasons for Exclusion

Articles were initially excluded based on reading the titles and abstracts, and then, for remaining articles, the full paper was evaluated. If an included article was identified as concentrating on public health systems, hospitals, or health care professionals, it was classified as such and only included in the study if it pertained to hospital resilience. *Public health system resilience* refers to elements that reflect broader choices made by the health system, such as media, supply chains, and nonpharmaceutical interventions; *hospital resilience* refers to choices made by and within individual hospitals; and *health care professional resilience* refers to the individual and group resilience of health care staff, such as psychological issues, physical injuries, or exhaustion experienced by staff. There was a significant amount of overlap; therefore, many studies were identified as belonging to more than one category (see Multimedia Appendix 3).

### Critical Appraisal of Individual Sources of Evidence

Two authors (JS and RH) used MAXQDA 2020 (VERBI Software), a qualitative data analysis tool, to code the data using a coding tree consisting of 7 larger categories, including governance, human resources, professional values, finance, security, planning and management, communication, background (pre-existing policies), and two other coding categories to map methods (including methodological limitations) and the dimensions included in our conceptual framework. A separate category for professional opinions, recommendations, and other cited articles was included to facilitate the synthesis. The quality of studies was not evaluated although we did include information on the type of study design, data collection methods, potential limitations, a summary of the results, main findings, and recommendations given within the articles (Multimedia Appendix 3).

### Data Synthesis and the Conceptual Framework

Results from the Chinese and English articles were initially synthesized separately by JS and RH, respectively; then, the two syntheses were combined by all authors. We synthesized the literature according to the Ridde et al [18] definition of health care system resilience: “the capacities of dimensions/components of a health system faced with shocks, challenges/stress or destabilizing chronic tensions (unexpected or expected, sudden or insidious, internal or external to the system), to absorb, adapt and/or transform in order to maintain and/or improve access (for all) to comprehensive, relevant and quality health care and services without pushing patients into poverty.”

**Figure 1:**
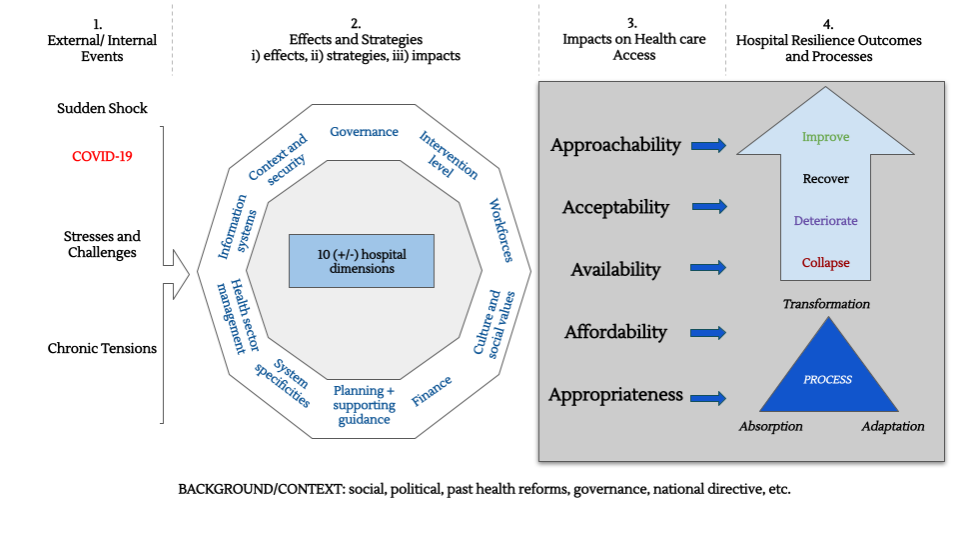
Resilience framework.

The synthesis of the articles was performed in terms of context, strategy, and impact. First, we explain the context in which a specific strategy is adopted, including the events in question and the effects of the pandemic experienced by the hospital in question. We then provide a synthesis of the strategies used, giving examples if necessary. Finally, we note the impacts of these strategies on health care access, which can theoretically be positive, negative, or neutral. The causality attributed to certain interventions is examined cautiously in the Discussion section. The right side of this resilience framework (Figure 1, parts 3 and 4) is not used in the evidence synthesis but will be examined briefly in the Discussion section.

This framework also helps us to address the question of how hospitals anticipate or react to crises. The *effect-strategy-impact* stage can illustrate different configurations:

- A reaction: When all three factors are present (an effect is felt, a strategy is adopted, and this strategy has positive or negative impacts)
- Anticipation: When strategies have an impact before a shock or are preventing a shock
- Inaction: When a shock has negative effects, but there are no strategies in place to react to this

The framework identified 10 conceptual dimensions of health systems: governance, intervention level, workforce, culture and social values, finance, planning and supported guidance, systems specificities, health sector management, information systems, context, and security, which we integrate into three larger categories with which to perform the synthesis: (1) human resources, (2) management and communication, and (3) the hygiene-security-planning nexus.

## Results

### Overview

**Figure 2:**
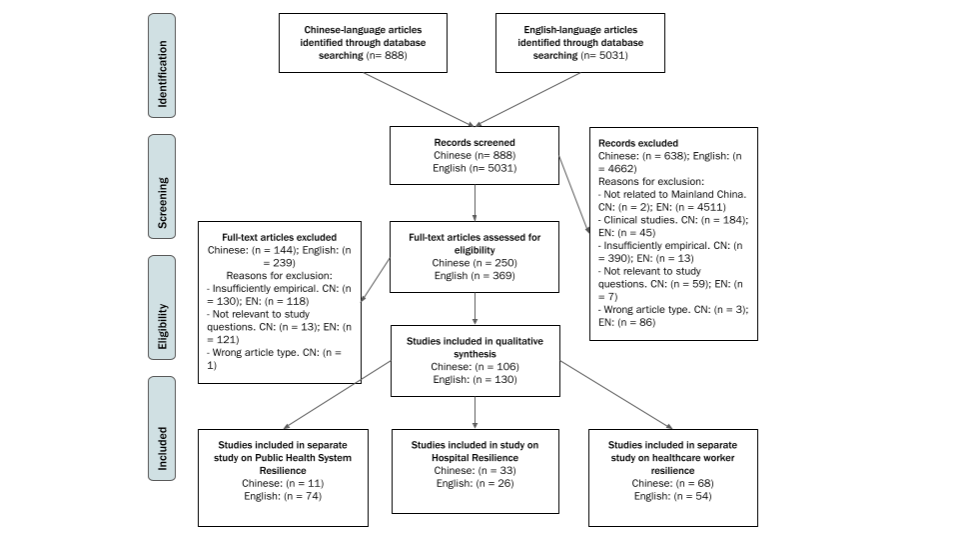
PRISMA (Preferred Reporting Items for Systematic Reviews and Meta-Analyses) flowchart. CN: Chinese; EN: English.

As shown in Figure 2, we obtained 888 articles in Chinese and 5031 in English.

We identified 236 studies that met the criteria regarding resilience in general, of which 59 studies, 26 in English and 33 in Chinese, met the criteria for inclusion in the hospital-focused study; Figure 2 shows the process of study inclusion in this scoping review. We mapped the distribution of study design according to region, type of study, category of hospital, and language (Multimedia Appendix 4).

**Figure 3:**
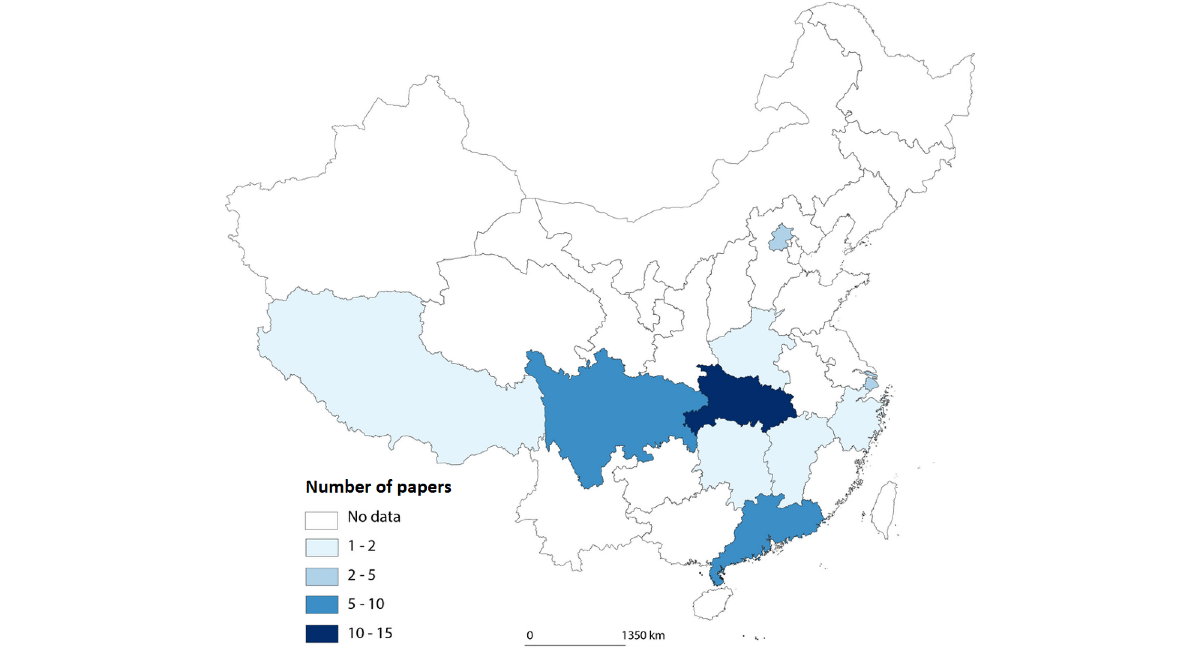
Geographical distribution of papers.

The geographical distribution of the papers is described in Figure 3; the studies were based on research undertaken at a diverse array of hospitals and settings. Only 2 articles were based on national surveys and therefore not focused on a single hospital. Understandably, the most represented geographical location with 14 papers of 59 (24%) was Wuhan in Hubei Province, with Sichuan Province in second place (n=8, 14%), followed by Guangdong (n=7, 12%) and Shanghai (n=5, 9%). A total of 50 (85%) studies were focused on tertiary A hospitals, the highest-ranked large hospitals in the country; 8 (14%) studies included various hospitals, including secondary hospitals, while only 1 study targeted primary health care providers.

Our analysis revealed that 94% (n=56) of the articles were explicitly identified as peer-reviewed articles, with 1 review article, 1 commentary article, and 1 short report. In terms of methodology, the studies were dominated by single case studies using mixed methods (n=30, 51%) and descriptive quantitative studies (n=22, 37%). There were 4 (7%) qualitative studies, 2 (3%) studies using other mixed methods, and 1 randomized study. The dimensions of hospital resilience most commonly referred to were health sector management (n=44), context and security (n=47), intervention level (n=8), planning and support (n=29), system specifics (n=8), information systems (n=8), workforce (n=31), and cultural and social values (n=4). Other dimensions such as governance and finance were not covered in the selected articles.

In terms of MMAT criteria, 85% (n=50) of articles contained clear questions and objectives, and addressed them appropriately. Quantitative studies adhered to the MMAT criteria to mixed degrees: the sampling strategies were often not made explicit (n=4) and many studies used some form of convenience sampling (n=5) due to accessibility and need for timeliness given the crisis context. It was often unclear whether the study was representative of the population (n=8), and in some articles (n=6), there appeared to be some overrepresentation of certain groups within the population (women and nurses in particular).

All quantitative articles were deemed to have used appropriate measurement tools, and only 9% (n=2) of the quantitative articles did not specifically mention response rates. Similarly, 7% (n=2) of the mixed methods articles were considered to have used an unclear methodology, and 17% (n=5) were identified as not integrating qualitative and quantitative data in a relevant manner. Only 36% (n=11) of articles explicitly considered limitations of the study methodology.

Studies published in Chinese were more likely to be case studies (n=21, 62%) compared to studies published in English (n=7, 27%). Chinese-language studies were also less likely to consider limitations of the methods used, with 71% (n=24) not mentioning any limitations, as opposed to 23% (n=6) of the English-language papers. Our MMAT evaluation determined that the papers published in English conformed more closely to the MMAT framework than those in Chinese.

We identified 10 different categories in which strategies to address the pandemic were used and 29 specific strategies recommended by the articles (Multimedia Appendix 5), with a description of the strategies involved and in which studies these strategies were mentioned.

Multimedia Appendix 5 illustrates that a number of different strategies were identified in the scoping review procedure, and the results of these are explored in the following synthesis.

### Human Resources

#### Reinforcements

After the initial outbreak of COVID-19 in Wuhan, the Chinese authorities made the decision to send personnel reinforcements from all over the country to Hubei Province to fight the epidemic. Wuhan is medically well equipped (9.25 hospital beds per 1000 inhabitants in 2018) and has 110,000 health professionals, including 40,000 medical practitioners and 54,434 nurses [19], but at the end of January 2020, reinforcements were nevertheless sent from all over the country. A total of 42,000 new health workers arrived in Hubei Province, including 35,000 arriving in the city of Wuhan, remaining in the area for between 18 and 50 days [20]. The implementation of a rapid response mechanism to the pandemic thus required the rapid integration of external reinforcements, as well as adaptation to the local hospital environment and management style [21]. Several articles reported the strategies implemented by hospitals to facilitate the integration of reinforcements and improve work efficiency [22,23].

- Strategy 1: Standardization of nursing procedures. In one hospital in Wuhan, the 27 nurses in a ward dedicated to patients with COVID-19 had all come from different departments (eg, infectiology and cardiology) from six different hospitals in Sichuan Province. These nurses had different experiences, skills, and habits; therefore, to facilitate collaboration and improve work efficiency, the management of this hospital introduced a new work system, standardizing nursing procedures and responsibilities of each staff member [24].
- Strategy 2: Creation of backup teams. This involved forming a team composed of staff members and external reinforcements in preparation for increased staff demand in COVID-19 infection wards and to compensate for a reduction in staff numbers due to infection. These teams were often formed strategically; for example, Jinyintan Hospital in Wuhan deliberately split nursing teams into teams comprising of backup (nonlocal) nurses and local nurses, experienced nurses and newly graduated nurses, and intensive care unit nurses and nonintensive care unit nurses to share experience, skills, and awareness of procedure [25].
- Strategy 3: Delineating the responsibilities of each staff member. Many hospitals instituted measures such as checklist interventions [26], training, and management strategies to ensure that the roles and responsibilities of all staff, especially transdisciplinary nurses and new staff, were clearly defined and that staff were aware of any changes. These new responsibilities included recording electrocardiogram results, organizing ward supplies, and observing critical patients [27]. Training interventions involved training on protective measures and the operation of medical equipment. As the backup nurses were not familiar with emergency equipment and instruments, hospitals set up a series of training programs to help the nurses understand the operation of various pieces of equipment [24].

#### Impacts

The articles reported many positive outcomes as a result of these interventions, including how the efforts helped facilitate the integration of reinforcements into the service and deliver quality care efficiently while maintaining the mental and psychological health of reinforcement staff [21,25]. For example, Feng et al [24] described how, between January 27 and March 15, 2020, as a result of the close collaboration between local nurses and backup nurses, 84 of 97 (87%) patients with COVID-19 admitted to the hospital were discharged, and the hospital only had one fatality. Care and psychological support offered to both backup and local nurses was timely, with none of their nurses reporting any severe psychological problems. Additionally, as Liu et al [27] note, reinforcement staff experienced negligible levels of infection, likely due to strict adherence to procedures, ample access to personal protective equipment (PPE), and special accommodations away from their families and other potential sources of infection.

### eHealth, Telemedicine, and Use of Technology

#### Context

Due to the contagiousness of SARS-CoV-2, new ways of caring for patients were implemented to reduce the risk of contamination. Family visits were restricted, and the loneliness of inpatients became a substantial challenge requiring hospital staff to pay more attention to the mental health of patients. To prevent and control the spread of the virus and to avoid cross-contamination, some departments closed their ambulance services and stopped receiving patients, while lockdown and the accompanying transport control measures made it difficult for non–COVID-19 patients to travel and receive treatment [28]. Due to these disruptions, many patients receiving radiotherapy, chemotherapy, or dialysis could not be treated in a timely manner [29].

To continue providing health care to the community and fulfill their obligations to patients, hospitals had to use other methods to provide care; therefore, a common element examined in the chosen studies was the use of telemedicine interventions. Telemedicine interventions serve the role of allowing patients to receive medical appointments, services, and treatment without having to visit a hospital; preparing and screening patients before they arrive at the hospital to facilitate their entry into the hospital and avoid contamination; monitoring patients with COVID-19 in home quarantine; and using human resources more efficiently. Additionally, mobile and digital technology was used by hospitals across China in a range of other ways to increase efficiency and reduce person-to-person contacts [30].

- Strategy 1: Use of online services for psychological issues in the population. A hospital in Chengdu implemented a multitiered intervention program, with online media, free hotline consultation, and targeted online video interventions provided to citizens with psychological problems, with crisis intervention provided on site [31,32].
- Strategy 2: Development of online screening mechanism for potential patients. A range of strategies were suggested to provide web-based consultations, appointments, prescription services and drug delivery, and other services, as a complement to in-person hospital services. For example, in a qualitative study of patients’ experiences with online services offered to non–COVID-19 patients, one patient reported: “Use of mobile apps in this pandemic is very important. You can pay, register, and view results on your mobile phone. You don't need to queue up at the outpatient clinic, and you can chat with a doctor online after you get home, so it’s far more secure” [33].
- Strategy 3: Using online platforms to monitor patients with COVID-19. A number of articles described a process of offering e-counselling support to patients who were struggling with the physical and psychological effects of the disease [34]. In addition to providing a greater monitoring and awareness of the individual patients, this intervention also allowed the staff to collect data to use in the improvement of in-hospital treatment [35-37].
- Strategy 4: Developing and using onsite information technology services and infrastructure. The use of nonmedical technology to improve hospital services, such as using app-based QR Codes (a machine-readable optical label, similar to a barcode) to share information and using robots for certain tasks to avoid person-to-person contact, was expanded during the pandemic [30,38].

#### Impacts

Telemedicine interventions were reported as an effective substitute or complement to onsite health care [35-37] and were also cited as being popular and time-saving among the majority of users [32]. The introduction of teleconsultations also reduced the difficulties of patients with chronic illnesses regarding the management and purchase of medicines [28]. Furthermore, the implementation of eHealth interventions allowed staff to address the needs of a higher number of patients while also helping to spread understanding regarding the virus risks and public health knowledge [39].

### Organization of Work and Health Care Worker Well-being

#### Context

At the beginning of the epidemic, medical personnel experienced panic and fear due to insufficient knowledge of the epidemiological characteristics of the virus and the need for protection, and many experienced a temporary shortage of medical supplies [40]. The problems of work overload were also highlighted in many papers, particularly the problems associated with new unfamiliar tasks for which nurses had not been specially trained [41], high work intensity [42], disrupted circadian rhythms, and restrictions and challenges of protective clothing.

These factors intensified workload pressures and led to anxiety, insomnia, depression, pain, symptoms of posttraumatic stress disorder, and grief. Furthermore, a higher workload led to worse hygiene behavior, such as reduced adherence to handwashing guidelines [32]. As 1 article explains:

high risk of professional exposure, the intense workload, the sharing of the patients’ anxiety, a feeling of helplessness while struggling to treat severely ill patients and many other factors can lead to high levels of psychological pressure, low confidence in one’s own work and depression among nurses, which affects their quality of work and their physical and mental health [24]

- Strategy 1: Readjustment of health care staff schedules. For health care staff who had direct contact with patients with COVID-19, a 4- to 6-hour schedule was implemented by a number of hospitals. Protection protocols in Chinese hospitals were extremely strict, especially for health care workers who were working directly with patients with COVID-19. Once PPE was applied, it was required that the wearer avoid all potential contamination risks, including physiological needs: eating, drinking, and using toilet [43]. The hospital thus reorganized the timetable of the staff, accounting for the physical needs of the staff, the efficient use of single-use protective material, and the needs of the patients. For example, in Jinyintan hospital (Wuhan, Hubei), three systems were successively implemented as early as December 2019: shifts of 4, 5, and 6 hours were tested, and after surveying staff perceptions of the three shifts, the hospital adopted the 5-hour per day system [25]. Another hospital in Wuhan implemented 6 shifts per day with a 4-hour rotation to allow nurses the time to take care of their physiological needs [23].
- Strategy 2: Increased flexibility of working hours according to the number and condition of inpatients. During the peak period of patient admissions, the number of staff was increased to provide an appropriate nurse-to-patient ratio, which is essential to ensure that patients receive appropriate care and that the workload of caregivers or staff remains reasonable. For example, in a hospital in Wuhan, each nurse was responsible for 6 to 8 patients [23]. The hospitals also used backup teams while using shorter shifts and appropriate working hours to reduce risks associated with workload, including lowered quality of work, medical errors, and increased rates of nosocomial transmission [25,32,41].
- Strategy 3: Providing material and psychological support to the staff. As well as ensuring provision of essential supplies, several hospitals provided high-nutrition meals to support staff and boost their immunity. Many strategies were used to provide psychological support, either through colleagues, health professionals, or specialized psychologists [44]. For example, a maternity ward in Tongji Hospital (Wuhan) established a WeChat group, “to promote scientific articles on mental health, to understand...problems in the life and work of the medical staff and subsequently to provide help and support...A psychological consultation platform was also established to provide medical staff a channel to vent their negative emotions and to offer psychological interventions when needed” [35].

#### Impact

These strategies improved working conditions for health care workers and quality of care for patients. A number of articles [45,46] highlighted that use of shorter shifts and appropriate working hours could be effective strategies to deal with mental health needs, work quality, and hygiene requirements. Furthermore, a flexible work schedule also meant that staff members were less affected by fatigue and stress. For example, in Tongji hospital in Wuhan, 97% (n=63) of the staff members were satisfied with the schedule of 5 hours per day, compared to 59% (n=37) satisfaction with the previous schedule of 6 hours per day [25].

### Management and Communication

#### Emergency Team and Nursing Management

##### Context

After the COVID-19 outbreak in Wuhan, China, human resources were rapidly reorganized within hospitals, between hospitals, and throughout the country. Transdisciplinary nurses without specific expertise in infectious diseases were brought in to support COVID-19 wards [3], backup teams were introduced, and frontline nurses had major changes in their responsibilities. However, many issues arose from this reorganization, such as nurses lacking understanding about their specific responsibilities [39]. Several strategies in management were used to increase the effectiveness of medical staff under these new circumstances:

- Strategy 1: Creation of new teams. Soon after the epidemic was declared, many new teams were created, such as the nursing technical support team, comprised largely of head nurses from different departments [45], and the emergency management and sensing control team, with a focus on procuring new information about the virus, and establishing an emergency management plan [22].
- Strategy 2: Implementation of a plan-do-check-act (PDCA) cycle, a management tool. This consisted of a repeated four-stage model for continuous improvement in quality management [46]. In terms of human resource management, this included 3 relevant components: defining the staff’s role and responsibilities, establishing a clear staffing structure and changing the shift handover modes, and testing and verification of procedures, such as evaluating nursing staff with questionnaires.
- Strategy 3: Implementation of regular training for health care workers. Given the speed of SARS-CoV-2 spread, health care staff required rapid training to properly apply the protection protocols and needed continuous information regarding the evolution of knowledge about the virus. In addition, reinforcements who were unfamiliar with the workplace also needed to familiarize themselves with their new colleagues and the work environment. Many hospitals in our study implemented a dual training system including online training and face-to-face training on topics such as “COVID-19 hospital infection prevention and control, hospital air purification management specifications, medical institution disinfection technical specifications, and personal protection requirements for disinfection and isolation” [41] was undertaken to improve care and reduce the risks of contamination between colleagues. The training content included the following elements: the characteristics of the service and the environment, spatial planning and reorganization, disinfection measures and knowledge of protection protocols, work procedures, and the use of medical equipment. As well as training, WeChat groups were established to communicate up-to-date information on the progression of the pandemic and knowledge of treatment options [30].

##### Impacts

Several articles quantitatively measured the effectiveness of different aspects of management interventions, finding that they succeeded in making staff aware of their roles and responsibilities, as well as clarifying the staffing structure and handover procedures [22,27]. Through training interventions and communication facilitated by the WeChat groups, frontline caregivers developed a better knowledge of the virus, which helped to alleviate their anxiety and fear [21,47], and they were better able to apply the protection protocols [48]. A quantitative study focusing solely on a training intervention on COVID-19 knowledge and training techniques provides an example of this, finding strong positive effects of the intervention on employee knowledge [40] and concluding that interactive simulation training is complementary to didactic teaching. In a hospital in Beijing, 7 days after the implementation of the standardized training program for 1125 medical staff, scores in a test on the prevention and control of nosocomial infection rose from an average of 69 of 100 to 88 of 100. The correct answers by supervisors rose from an average of 83 of 100 to 92 of 100 (n=309), while the proportion of staff members wearing surgical masks increased from 86% to 93%, and the proportion of adherence to hand hygiene protocols increased from 92% to 96% (n=1630) [41]. In Chen et al [46] the PDCA team identified the problem of poorly defined responsibilities, noting that, although 12% (n=4) of a small nursing team initially lacked awareness of their responsibilities, this was reduced to 0% following a training intervention.

#### Communication and Information

##### Context

During the early stages of the outbreak, as knowledge of the virus rapidly evolved and the number of patients in the hospital increased daily, hospitals were required to react immediately to the situation and readjust strategies accordingly, whether in terms of protection protocol, patient care, or organization of work. The situation was more complex in hospitals with external reinforcement from other provinces because, according to one article, “each medical team has its own process and philosophy of care, the only way to provide quality care to patients is to coordinate and standardize and homogenize care” [47]. Quickly and accurately conveying expert information and the response plan to staff at all levels became a serious challenge for various medical institutions. In this situation, fluid communication between the different parties involved (government, hospital, carers, patients and families, etc) was essential.

- Strategy 1: Implementation of regular meetings between the different team members for daily briefings. For example, in Tongji Hospital in Wuhan where national medical aid teams served in a “whole-system-takeover model,” nursing department staffers worked in partnership to establish a range of measures including smoothing communication channels through daily meetings. According to one author: “In the early stages, we held daily nursing council meetings to shorten the adjustment period and standardize the work in order to shift from a ‘wartime state’ to a daily routine” [5].
- Strategy 2: Promotion of the use of new information and communication technologies to aid communication between colleagues. Use of communication platforms, usually WeChat groups, and occasionally telephone exchanges, was identified in a number of articles. In Tongji Hospital, to provide an effective communication and information mechanism, a WeChat group with all the nursing staff was set up to enable communication at any time. In addition, the hospital set up a daily nursing information system: the progress of nursing work as well as problems encountered in the quality control of care were analyzed and then sent to everyone in image/text form [5].
- Strategy 3: Promotion of the use of visual materials to better convey information to health care staff. This involved the use of physical signs such as multicolored arrows indicating the different hospital zones and posters of protection protocols displayed in different zones [21].

##### Impact

Some evidence in the articles indicates that the aforementioned communication measures were effective in improving the psychological health and efficiency of health care workers. In the People’s Hospital of Wuhan University, 1 week after a *visual management* communications intervention was implemented, the time taken to obtain materials was shortened, the satisfaction rate of medical staff improved, and nursing quality increased [21]. Moreover, regular updates on the state of scientific understanding of the virus and informing the staff promptly about key information using the framework of *what we know*, *what we don’t know yet*, *what we have* (in the hospital), *what we don’t have* (in the hospital), and *what we are doing* was seen as particularly effective, both in relieving the anxiety of health care workers and improving the effectiveness of protection measures [41,47,48].

### Security, Hygiene, and Planning

As well as analyzing the number of infected staff, a large number of articles in the scoping review examined the reasons for infection of health care workers and presented hospital strategies to reduce the risk of nosocomial infections.

#### Protection Protocols (Change and Application of Protocols)

##### Context

The issue of contamination risk is one of the most frequently discussed topics in the articles and relates to many dimensions of hospital resilience, such as human resources, management, communications, and information. The risk of nosocomial infection was extremely high in Wuhan, especially in the early phase of the outbreak. One study found that 84.5% (1426/1688) infected health personnel believed that their infection had been acquired in the hospital wards [49]. To reduce the contamination risk as much as possible, Chinese hospitals implemented strict protocols for hospital admissions, discharge procedures, PPE, and the application of social distancing rules.

- Strategy 1: Strict management of hospital space. Access to the hospital analyzed in Lu et al [50] was closely controlled in terms of body temperature and mask wearing. Patients with a temperature over 37.5 °C or showing respiratory symptoms were redirected to the fever ward or the emergency department.
- Strategy 2: Focus on environmental contamination with routine disinfection. In the COVID-19 unit, strict measures were applied regarding the disinfection of medical instruments (stethoscopes, thermometers, etc). This was described in detail in 1 article, which explained how floors, tables, chairs, and diagnostic and treatment beds were wiped and disinfected regularly with 1000 mg/L of chlorine disinfectant and that this behavior was regularly monitored [29].
- Strategy 3: Encouraging health care workers to apply personal equipment protocols appropriately, according to their role and their level of contact with patients with COVID-19. To help staff to properly apply the protocols, hospitals proposed regular training for staff and the establishment of a 24-hour supervisor position to verify the appropriate application of protocols when entering and leaving the buffer zone. Hospitals often introduced comprehensive management plans involving screening, personnel management, disinfection and hygiene procedures, and training and supervision of employees, as well as PPE supply chains.
- Strategy 4: Restricting family visits to avoid patient-family contact. Family visits were restricted, as they increased the risk for nosocomial transmission; however, many hospitals implemented a video visit system to facilitate exchanges between patients and their families. One article quoted a staff member: “For people who come to the hospital to visit patients, the warden enables the video visit with an iPad connected to the nurses’ iPad at the patient’s bedside, which enables exchange with the visitors” [51].

##### Impact

Only a few articles evaluated the impact of these strategies on infection rates, with most concluding that no medical staff member was contaminated by SARS-CoV-2 during this period. With regards to PPE use, a regression analysis in self-reported compliance with security protocols [32] found two seemingly contradictory findings: although staff in high-risk departments have higher rates of compliance with security protocols, further contact with at-risk patients had a negative effect on compliance. The research did not capture information to determine whether this was due to resource shortages, human deficiency, high workload, or other factors, but reducing workload through reinforcements, ensuring resource supply and increased training is recommended as an intervention. In terms of environmental contamination, 1 article [52] found the highest rates of environmental contamination in the isolation ward for pregnant women, even when compared to the fever clinic. It was hypothesized that this was due to the differences in ventilation and the number of visitors. This article also found that hand sanitizer dispensers and used gloves were greater sources of contamination than eye protection or face shields.

#### Personal Protective Equipment

##### Context

In the response to the COVID-19 outbreak, the supply of PPE was a substantial challenge globally. This problem was also present in China, where several regions had a shortage of PPE and disinfection products [40]. Hospitals were required to adjust the variety and quantity of protective materials in a timely manner to find an ideal balance between the level of equipment consumption and storage capacity, which was essential to ensure continuity of care.

- Strategy 1: Implementation of an inventory register for important materials while standardizing the process of managing and using these materials. In many hospitals, a physical security team leader was put in charge of recording the real-time use of equipment and strictly controlling the receipt and distribution of materials [35].
- Strategy 2: Avoiding overconsumption and waste of materials. The presence of the hygiene team and the supervisor in the application of caregiver protocols was to make sure that staff wore PPE correctly and avoided PPE overuse [53]. In another hospital, when restocking materials, it was not permitted to mix materials with different expiry dates to ensure that different materials were used in order of expiry dates, from oldest to newest, to avoid waste [21].
- Strategy 3: Decrease in the protection level for the provision of certain non–COVID-19 services in view of the shortage of medical resources. Some studies examined decreased protection measures to identify the minimal level of protection needed in different hospital areas. For example, a study in the Huaxi Hospital of Sichuan University suggested that the staff in non–COVID-19 intensive care units did not need to wear full body protective overalls, thus saving on PPE [54].

##### Impacts

Overconsumption of PPE was a common problem in hospitals, particularly in the early and midstages of the outbreak. However, according to surveys from a hospital in Shenyang, the aforementioned strategies contributed to the optimization of PPE and disinfection supplies, allocating based on needs and stock while ensuring that frontline personnel were well protected [40]. The Huaxi Hospital of Sichuan University applied these protective protocols to its pediatric intensive care unit, finding that health care professionals (91 people) and household members (5 people) in contact with COVID-19–positive patients wore only masks and did not wear the full protective suit required by some institutions, yet there were no infections in the ward [54], suggesting that lighter PPE could be sufficiently effective.

#### Reorganization of Services

##### Context

During the outbreak, hospitals had to reorganize their services to both increase capacity and reduce the risk of contamination. The changes in infrastructure, hospital procedure, and protocols in Chinese hospitals involved substantial changes. For example, a hospital in Wuhan revised 32 items on its regular hospital procedures to transform a general hospital into a designated COVID-19 treatment hospital [45], and a number of case studies were written on particular changes in procedure and ward renovation [37].

- Strategy 1: Transformation of non–COVID-19 hospital areas into specialized COVID-19 wards. Many hospitals lacked a negative pressure chamber to provide a buffer zone; therefore, many large non–COVID-19 hospital areas were required to be transformed into specialized COVID-19 wards to accommodate the growing number of patients with COVID-19 [53].
- Strategy 2: Reorganization of space. In designated COVID-19 hospitals, necessary infrastructure changes were implemented, which included setting up fever tents, ward renovation, unidirectional channels for patients, and converting sections of the hospital for patients with COVID-19. These were done to minimize contact between infected and uninfected individuals, reduce patient flow throughout the hospital, and maximize the shared space available to patients with COVID-19. Another example is the creation of the “three zones and two passages” system (Chinese: 三区两通道) that included a contaminated zone, potentially contaminated zone, and a clean zone, as well as two separate passages for medical staff and patients.
- Strategy 3: Reorganization of inpatient rooms. Certain hospitals decided to convert double rooms into single rooms, whereas for hospitals that were forced to put several patients in the same room, a distance of more than 1 meter between beds was maintained.

##### Impacts

The impacts of these strategies were not examined in detail in the included studies. Gao et al [55] claimed that their management strategy contributed to effective prevention of virus spread in the endoscopy center in Sichuan; however, none of the articles claimed to provide strong evidence of the effectiveness of a given intervention.

## Discussion

### Summary of Evidence

In this scoping review, we identified 59 studies that addressed resilience in hospital settings across China in the context of the initial SARS-CoV-2 outbreak in the first half of 2020. Our findings indicate a wealth of research describing certain strategies used to improve hospital resilience, particularly those concerning human resources: management, communication, security, hygiene, and planning. We found that much attention was focused on training, health care worker well-being interventions, eHealth and other technology-related interventions, and work organization interventions, while training and management interventions were also subject to more rigorous quantitative analysis. Some themes, such as information systems and reinforcements, were mentioned in a small number of studies and lacked rigorous analysis, while others, such as hospital financing and the development of new health care infrastructure, were neglected in the literature despite being mentioned explicitly in Chinese official policy papers [56]. Most importantly, our findings also represented a paucity of rigorous research focusing on the effectiveness of interventions and a lack of research attempting to unify these different elements within a resilience framework.

In terms of the “Effects—Strategies—Impacts” framework, there were some cases of inaction, anticipation, and reaction represented in the literature. Only a handful of studies examined cases of inaction; for example, Gao et al [49] analyzed the reasons for personnel infections in the early stages of the outbreak. Most studies referred to actions taken in anticipation of major outbreaks in provinces with only limited spread. Some studies, especially those carried out within Wuhan, described a strong reaction to a serious ongoing outbreak.

The majority of the included studies provided details on the effects and strategies with an appropriate methodology, whether quantitative, qualitative, or mixed. However, few studies performed any kind of systematic analysis to evaluate the impacts of these strategies and were more descriptive in nature. The goal of a large portion of the studies was to share knowledge as quickly as possible, but the lack of rigorous analyses provides issues in identifying effective strategies. An important characteristic in the interpretation of a strategy or a specific intervention was that most studies were written by health care workers working directly in a given hospital during the outbreak. Participation by health care workers in the process of knowledge creation can be an invaluable tool, demonstrating what Alexander et al [57] have identified as “reflexivity on action,” and enabling the creation of a “collective space for health professionals to reflect on and improve their practices.” However, this process could also represent a bias that can bring into question the neutrality of the scientific research process, especially as many articles, particularly those in Chinese, did not consider the methodological limitations.

Similarly, as China’s research and medical communities are not independent from politics [20], political factors may have played a role in the choice of papers written and published, potentially neglecting those that found negative results. These two factors, politics and the predominance of health care workers, as opposed to professional researchers as authors, may also have limited the scope of articles concerning resilience issues such as finance or power structures, which can be sensitive and politicized. Many of the articles that examined hospital strategies to address health care worker health issues emphasized the physical and mental health of nurses while often neglecting the issues faced by other health care providers, including doctors. One possible reason for this phenomenon is that doctors may have more difficulty discussing problems encountered in work and sharing mental health concerns with colleagues [58]. Similarly, gender issues and potential inequalities were not discussed in the selected papers. Despite significant gender gaps existing in health care professions—men being overrepresented in senior health care roles and underrepresented in nursing staff—this was not considered in the selected articles. This suggests that a “gender blindness,” the systemic failure to acknowledge gender differences in health [59], may be present in the case of Chinese hospitals.

These papers also highlighted how some processes undertaken during the pandemic attempted to increase health care access in ways that could potentially lead to a positive transformation process (as mentioned in the resilience framework). For example, articles focusing on eHealth and *internet hospital* interventions [39] mentioned ways that the transition to telemedicine provoked by the SARS-CoV-2 pandemic could be used to make health care more approachable and affordable, and improve availability to vulnerable groups across the country. Further research is needed to examine whether these resilience processes could lead to improved access to health care in China’s hospitals following the pandemic.

### Recommendations for Health Care Practitioners and Managers

There are a number of recommendations offered to health care practitioners within the articles (Multimedia Appendix 5). Improving patient awareness of online services enabled patients to better respond to these public health emergencies and reduced unnecessary round trips between home and hospital [41,50]. Artificial intelligence and internet technologies can be used for online self-assessment systems, robots can be used in guiding patients and delivering medicines within the hospital, and QR Codes can be used for collecting patient and visitor information [46]. Studies also found that China’s advanced use of technology has a crucial role in many elements of a resilience framework, including training, knowledge management, and transfer and information systems. However, it is important to note that these recommendations were not substantiated by rigorous evidence. For example, Yan et al [30] provide a descriptive examination of information system strategies used by China’s most reputable hospitals and offer recommendations without any demonstration of evidence to support the recommendations. In terms of nursing management, clear role recognition is seen as an important prerequisite for better practice. Nurses in 1 article [3] criticized the ambiguity of the roles given to over half of transdisciplinary nurses, suggesting that “more detailed role classification, clearer role definitions and job descriptions, and appropriate suggestions for expanded responsibilities would be effective methods to alleviate role ambiguity and improve work efficiency.” Other articles suggested that role ambiguity can be remedied with fairly simple interventions, such as a PDCA cycle to improve standardized nursing management in an intensive care unit ward [46].

With articles that analyzed the impact of training interventions, both more traditional and online training interventions were associated with positive effects on knowledge and behavior of staff regarding safety procedures, when compared to results before the training. Online or massive open online course–based training is an appealing alternative to in-person training when infection risk reduction is a relevant concern. To build hospital resilience, articles argue that staff training for outbreak and infectious disease practices should continue in regions without ongoing outbreaks [49] and should continue after the outbreak has subsided [46]. We can conclude that the ability to provide timely, effective training interventions in response to a health care emergency is a crucial element of a resilience framework.

Infection control measures comprise a crucial element of hospital resilience and many recommendations were given, despite not always being supported by data. Li et al [32] recommended targeting certain infection control interventions in low-risk departments, as there may be higher risk to staff on other wards (eg, the maternity unit) compared to the infectious disease unit due to disparate security measures and PPE use. Many articles related to infection control and environmental contamination recommended using risk-averse strategies with multiple layers of redundancy to reduce the risk of nosocomial and health care worker infection. Xu et al [60] also recommended that medical institutions should implement ward reconstruction so that nonspecialized hospital buildings are able to meet the requirements of an infectious disease unit.

### Recommendations for Researchers

The results of these studies demonstrate the degree to which China’s health care system responded and adapted to the outbreak through several innovative measures. Although evidence of the effectiveness of certain interventions was not provided, the collection of studies from across hospitals in China offers strategies that, together, have likely contributed to the decrease in daily nosocomial infections from a peak of 127 new health care worker infections on January 23, 2020, to the first day with 0 new cases on March 8, 2020 [61].

Due to the short time frame, the lack of academic diversity in the research areas, political concerns, and publication bias, this scoping review highlights the need for more rigorous intervention research and evaluation, and the inclusion of multidisciplinary teams involving social science researchers and data scientists [62]. Gilson et al [63] have called for a structured research agenda to inform health policy and system responses to COVID-19, which should include resilience research in China’s hospitals.

This scoping review was not intended to draw conclusions about the causality of any particular strategy; therefore, a *realist review* [64] would be a useful way of determining middle-range theories specific to the resilience of China’s hospitals in the outbreak.

Our research also revealed that there are relatively few articles that have used the concept of resilience in a Chinese medical context, indicating that China’s hospitals do not consider a resilience framework as part of their research. Despite increased use in academic and professional contexts, the popular concept of *health systems resilience* has not yet reached conceptual maturity [62], and according to a recent scoping review, “empirical studies fundamentally differ in the way that resilience is understood in a healthcare context” [65]. In China, the multiple possible translations for the term *resilience*—most prominently, *tanxing* (弹性), *renxing* (韧性), and *fuyuanli* (复原力)—three terms with subtly different connotations, demonstrate this lack of conceptual clarity. Further research needs to be undertaken to understand how the concept of resilience translates and is understood across cultures and academic contexts.

Another element that must be addressed is the trade-offs associated with the risk-averse strategy used in Chinese hospitals. Some studies noted that hospitals chose to implement a highly risk-averse strategy and that this did not allow them to determine what the minimal effective level of PPE use was to maintain effective protection [27], which poses problems for knowledge transfer to regions or situations with more limited capacity or resources. As Jin et al [52] notes “...(L)ack of evidence means we are using a precautionary approach which often results in our applying all available controls all the time.” Future comparative work could clarify whether China’s successes could be replicated without such extreme levels of personal protection or whether a highly risk-averse *zero-tolerance* policy for nosocomial infection is the optimal choice.

Financial constraints, which comprise a central aspect of health systems resilience, have also been understudied in the Chinese context. Although comparative studies have examined macro-level decisions and cost-benefit trade-offs in COVID-19 policy between countries including China [66], our study found a lack of research pertaining to financial constraints faced by Chinese hospitals and other relevant decisions at the hospital level.

### Limitations

We were unable to perform a risk-of-bias test for this paper; therefore, the issues resulting from political or other biases were difficult to determine. As few of the articles were written by non-Chinese citizens or were peer reviewed by external reviewers, selection effects caused by censorship cannot be excluded.

The exclusion criteria we chose meant that we did not include gray literature in this review, but it is worth noting that media articles, social media content, and government white papers may also provide relevant sources of information that may help better understand how Chinese hospitals have sustained resilience during the SARS-CoV-2 outbreak. Additionally, we only included articles released soon after the outbreak; therefore, articles conducted later in 2020 and in 2021 related to similar topics may have reached different conclusions [67].

### Conclusion

Our scoping review demonstrates that there is a wide range of studies concerning hospital resilience in the Chinese context and that this literature helps us to understand the strategies used by the hospitals in China during the SARS-CoV-2 outbreak. The literature, both in Chinese and English, can provide important lessons on reinforcements, organization of work, eHealth, telemedicine and use of technology, health care worker well-being, emergency team and nursing management, training, communication and information, protection protocols, PPE, and reorganization of services.

Although this review demonstrates that the evidence is generally insufficient to determine the effectiveness of specific strategies, some preliminary results on the effectiveness of training interventions, technology use, and management interventions, such as checklists and the PDCA cycle management, are provided. Furthermore, the study illuminates some common characteristics that have characterized what has generally been viewed as an effective strategy against the SARS-CoV-2 outbreak [66], including risk aversion and redundancy.

## Appendix

Multimedia Appendix 1 Search terms for English-language search.

Multimedia Appendix 2 Chinese-language search terms.

Multimedia Appendix 3 Mixed-Methods Appraisal Tool analysis.

Multimedia Appendix 4 Description of included studies.

Multimedia Appendix 5 Hospital resilience strategies and relevant papers.

Multimedia Appendix 6 PRISMA (Preferred Reporting Items for Systematic Reviews and Meta-Analyses) checklist.

## Abbreviations

CDC: Centers for Disease Control and Prevention
CNKI: China National Knowledge Infrastructure
MMAT: Mixed-Methods Appraisal Tool
PDCA: plan-do-check-act
PPE: personal protective equipment
PRISMA: Preferred Reporting Items for Systematic Reviews and Meta-Analyses
WHO: World Health Organization
